# The Nurses' Co-Operation

**Published:** 1920-01-17

**Authors:** 


### THE NURSES' CO-OPERATION.

The following statement from the members of the Nurses' Co-operation to the nurses on the nursing staff, dated 22 Langham Street, W. 1, December 29, 1919, and signed L. A. Crowe, Secretary of the Co-operation, has been circulated amongst the nurses :—

The members of the Co-operation have given careful consideration to certain complaints and requests which have been made by certain nurses with reference to the constitution and control of the Co-operation and the management of the Howard de Walden Home for Nurses.

With regard to the constitution and management of the Co-operation, the complaints and requests are as follows :

(1) That the nurses themselves have no say or control in the management of their own affairs.

(2) That the nurses should be allowed to become members of the Co-operation.

(3) That the Memorandum of Association of the Co-operation should be altered to provide that, in the event of the Co-operation being wound up, any surplus shall be divided amongst the nurses.

(4) That the Co-operation shall become co-operative, and the nurses themselves shall be sole parties to participate in and benefit from any surplus funds that can properly be said to be available for distribution.

The members of the Co-operation find difficulty in understanding the complaint that the nurses have no say or control in the management of the Co-operation. Under the regulations contained in the Articles of Association the affairs of the Co-operation, financial and otherwise, are controlled by the Committee of Management. This Committee is composed of ten representatives elected by the nurses and seven members of the Co-operation. Consequently, the nurses' representatives have a majority, and the nurses have control over the management of the Co-operation and the expenditure of its money for the benefit of the nurses within the scope of the powers of the Co-operation.

The funds of the Co-operation are derived from members' subscriptions, donations from outside sources, and the percentages retained of the fees earned by the nurses, which have formed the main source of income in recent years.

These funds have been used in meeting current expenditure (including the expense of the Sickness and Benefit Fund), and acquiring and equipping the offices at 22 Langham Street, and the Howard de Walden Home. It is due largely to the generosity of Lady Howard de Walden and other ladies and gentlemen who were members of, or interested in, the Co-operation in years gone by that we now own these valuable properties free from any mortgage. The unexpended balance when the accounts were last made up (December 31, 1918) was represented by cash and investments to the amount of about £3,000, which is none too large a reserve to hold against unexpected contingencies.

The nurses cannot fail to appreciate the advantages they derive from their association with an institution of this nature, which provides an organisation for securing employment and collecting their fees (and suing for them if necessary) and watching their interest, but itself makes no profit for its members.

The fact that the Co-operation, though registered under the Companies' Acts, has not the word "Limited" included in its name indicates to the public that no benefit can be derived by its members from the earnings of the nurses. The "Memorandum of Association" which had to be approved by the Board of Trade when permission was originally given to omit the word "Limited" expressly provides that no member may make any profit out of the business of the Co-operation, and that, if it is wound up, the assets may not be distributed amongst the members, but any surplus assets remaining after payment of liabilities shall be handed over to some other institution having similar objects, to be selected by the members or by a Judge of the High Court.

The members have taken legal advice upon the matter, and are advised that the claims that the nurses should be allowed to become members, and that, on winding up, the surplus assets should be divided amongst the nurses, cannot possibly be granted without a Special Act of Parliament. The members have decided that they will not themselves be party to any application for such an Act. They feel that such alterations would not be in the interests of the nurses, and would be directly opposed to the basic principle of the whole constitution of the Co-operation, which is that it has to be worked for the benefit of the nurses as a body. Nurses of past generations and original founders and contributors to the funds of the Co-operation have accumulated the present funds on this basis, but the granting of the claims now made would enable the staff at any particular time to wind up the company, sell all its assets, and divide the net proceeds amongst themselves, thus benefiting a certain number of individual nurses without any regard to the interests of future generations.

In order to place the nurses in a position for forming a judgment upon an issue so momentous for the very existence of the Co-operation, the members wish to know if the above-mentioned complaints and claims represent the views of the majority of nurses.

If it is found that a substantial majority of the nurses are in favour of these alterations which, as already explained, are both undesirable and illegal, then it will be clear that the nurses are entirely out of sympathy with the members, and the only course open to the latter will be to wind up the Co-operation and take the directions of the Court as to the disposal of the offices, the Home, and the other assets.

Every nurse on the staff is therefore most urgently desired to fill in answers to the question on the enclosed card, sign it, and return it to the Secretary at 22 Langham Street, on or before January 14, 1920. A stamped and addressed envelope is enclosed for the purpose.

It is hoped that every nurse, whatever her views may be, will comply with this request, and thus give the members some guidance.

